# Self-Medication Practices in Mekelle, Ethiopia

**DOI:** 10.1371/journal.pone.0097464

**Published:** 2014-05-12

**Authors:** Tadele Eticha, Kalkidan Mesfin

**Affiliations:** Department of Pharmacy, College of Health Sciences, Mekelle University, Mekelle, Ethiopia; Universidad de Valladolid, Spain

## Abstract

**Background:**

Self-medication makes consumers more health conscious, reduces treatment burden on healthcare facilities and curtails the cost and time of obtaining access to treatment. However, it increases risks such as drug resistance, adverse drug reactions, incorrect diagnosis, drug interactions and polypharmacy. The purpose of this study was to assess the practices and factors associated with self-medication in Mekelle, Tigray region, Ethiopia.

**Methods:**

A cross-sectional study was undertaken in Mekelle from February to March 2013. A structured and pre-tested questionnaire was used for data collection to assess self-medication practices. Data were analyzed using of Statistical Package for Social Sciences (SPSS) version 20.0.

**Results:**

Among self-medicated study participants, 199(73.7%) were males and 71(26.3%) were females with mean age of 28.65 years. The most frequently reported illnesses or symptoms of illnesses that prompted self-medication of study participants were headache/fever (20.7%), gastrointestinal diseases (17.3%) and respiratory tract infections (15.9%) with the main reasons being mildness of the disease, prior experience and less expensive. The majority of drug consumers made their requests by telling their symptoms, by mentioning specific names of the drugs and by showing old samples. Analgesics/antipyretics, gastrointestinal drugs, respiratory drugs and oral rehydration salt were the most frequently requested categories of drugs. Pharmacists followed by other healthcare providers were the most frequently reported source of drug information for self-medication.

**Conclusions:**

The results of this study demonstrated that self-medication practices were common for a wide range of illnesses. Health professionals, especially community pharmacists need to educate people on the benefits and risks of self-medication to encourage responsible self-medication.

## Introduction

Self-medication is an important part of daily self-care behavior and one of the vital issues under debate in healthcare systems [1]. According to World Health Organization, self-medication is the selection and use of medicines by individuals to treat self-recognized illnesses or symptoms [2]. The International Pharmaceutical Federation defines self-medication as the use of non-prescription medicines by people on their own initiative. Self-care, including self-medication, has been a feature of healthcare for many years and people have always been keen to accept more personal responsibility for their health status [3].

Self-medication is a fairly widespread practice in the world, particularly in economically deprived communities. When practiced correctly, self-medication has a positive impact on individual and healthcare system. It allows patients to take responsibility and build confidence to manage their own health, thereby, promoting self-empowerment. Furthermore, it can save the time spent in waiting for a doctor, and even save life in acute condition and may contribute to decrease healthcare cost [1].

The World Health Organization has also pointed out that responsible self-medication can help to prevent and treat ailments that do not require medical consultation and provides a cheaper alternative for treating common illnesses. Nevertheless, the individual bears primary responsibility for the use of self-medication products. All parties involved in self-medication should be aware of the benefits and risks of any self-medication product. Self-medication may be associated with certain risks such as drug resistance, drug interactions, adverse drug reactions, increased polypharmacy, incorrect diagnosis and drug dependence [1], [2], [4]–[8]. The present study was thus conducted to evaluate the practices of self-medication and to identify factors associated with it in Mekelle, Tigray region, Ethiopia.

## Materials and Methods

### Study Setting

The study site was Mekelle town, Ethiopia. Mekelle is the capital city of the Tigray Regional State, northern Ethiopia, situated at 783 km to the North of Addis Ababa.

This study was undertaken in community pharmacies found in Mekelle. There was a total of 11 community pharmacies, 37 drug shops and nine drug vendors in Mekelle town. Community pharmacies were classified into private and public community pharmacies. Three community pharmacies were selected randomly from each, and therefore, six community pharmacies were included in this study.

### Study Design, Participants and Sampling

A cross-sectional study was performed in the selected community pharmacies from February to March 2013. Participants in the survey were all people who came to the community pharmacies for self-medication. The sample size was calculated by considering 95% confidence interval, 5% margin of error and 10% contingency for loss. The calculated sample size depending on the previous study, which recorded 27.6% of the prevalence rate of self-medication in Jimma town, southwest Ethiopia [9] was 307. A convenient sampling technique was applied to distinguish the study participants from among those who came to the community pharmacies to buy drugs for self-medication. Study participants were interviewed after they made their requests, but before they were provided with information on the drugs they requested.

### Ethical Consideration

The study was approved for ethical issues by the Health Research Ethics Review Committee of College of Health Sciences, Mekelle University. Further approval was obtained from the community pharmacies before conducting the survey. Written informed consent was obtained from each respondent prior to the interview.

### Data Collection and Analysis

A structured questionnaire to rate self-medication practices and its determining factors was developed in English; then translated into the local language (Tigrigna) and back into English to check the accuracy by an independent translator. The standardized questionnaire had been pre-tested before the actual data collection. The questionnaire consisted of socio-demographic characteristics of study participants, types of illnesses or symptoms of illnesses for which self-medication was sought, reasons and type of requests for self-medication, sources of advice and category of drug products demanded for self-medication.

Data were introduced in the Statistical Package for Social Sciences (SPSS) version 20.0 to generate descriptive statistics. The results were shown in absolute figures and percentages as depicted in Tables and Figures.

## Results

Of the total of 307 questionnaires distributed to be filled during the survey period, among them 270 (87.9%) were filled completely and collected, which renders the response rate of 87.9% while questionnaires with incomplete information were rejected.

### Socio-demographic Characteristics

The mean age of the study participants was 28.65 years and most (42.6%) of them were in the age group of 25–35. The majority (73.7%) of the study participants were male. More than half (53.0%) of the respondents graduated from college or university while 85 (31.4%) had secondary school education. The majority (44.8%) of the respondents were students and 101 (37.4%) earned monthly household income of 500–1000 Ethiopian Birr (ETB, exchange rate 1 USD = 18.8273 ETB). Of the total self-medicated people, 22.6% had chronic disease(s), 7.8% and 5.2% were pregnant and breastfeeding mothers, respectively (Table 1).

**Table 1:** Socio-demographic characteristics of study participants (n = 270).

Characteristics		Frequency	Percentage
Gender	Female	71	26.3
	Male	199	73.7
Age	<25	105	38.9
	25–35	115	42.6
	>35	50	18.5
	Mean age	28.65	
Education status	Illiterate	21	7.8
	Primary school	21	7.8
	Secondary school	85	31.4
	Collage/university	143	53.0
Employment status	Employed	75	27.8
	Unemployed	49	18.1
	Student	121	44.8
	Merchant	25	9.3
Monthly income in ETB	<500	72	26.7
	500–1000	101	37.4
	>1000	97	35.9
Special drug consumers	Pregnant	21	7.8
	Breast-feeding	14	5.2
	Has chronic disease(s)	61	22.6

Exchange rate: 1 USD = 18.8 Ethiopian Birr (ETB).

### Self-medication Practice

**Figure 1:**
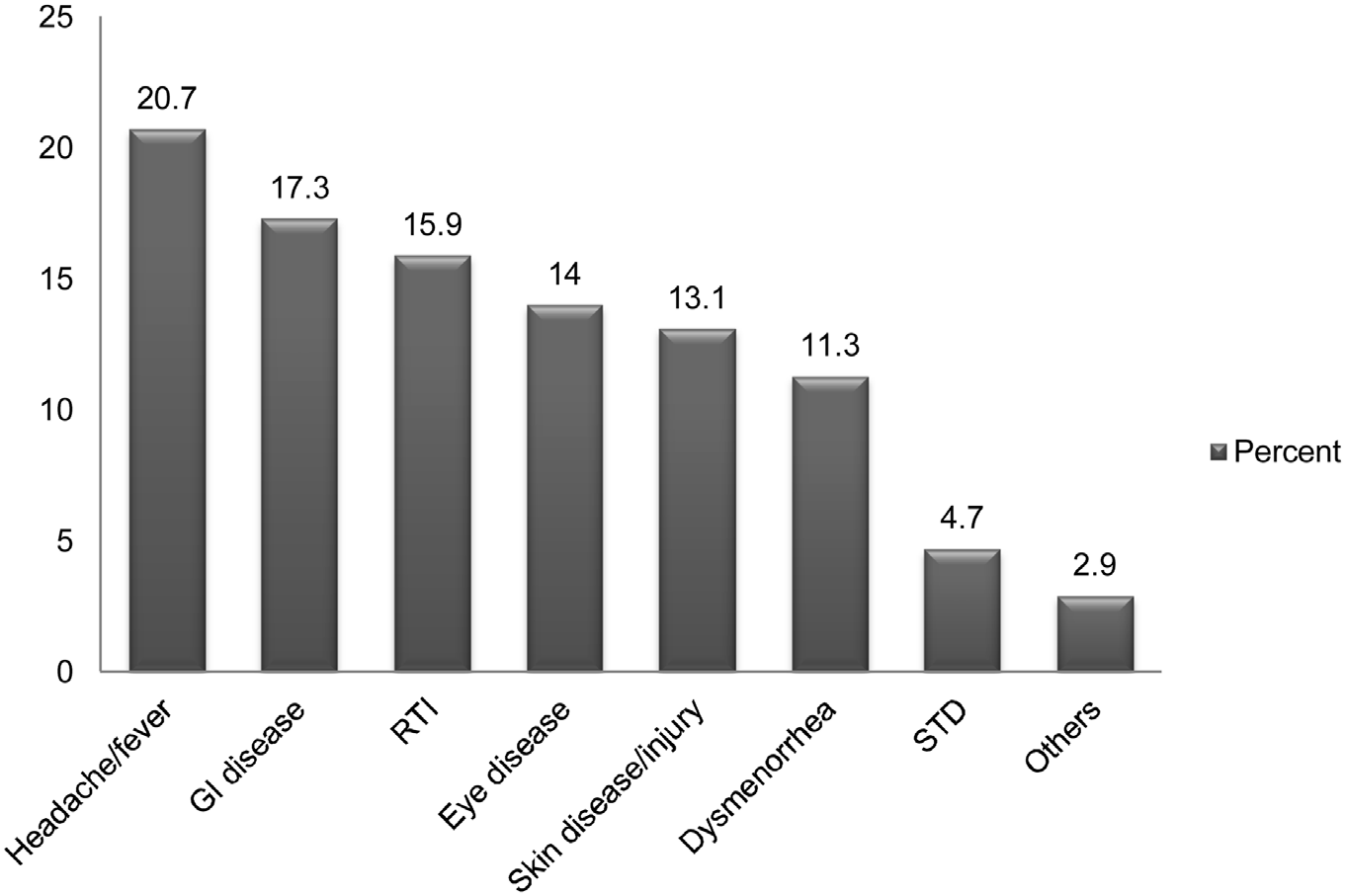
Type of illnesses reported by the study participants (n = 270). Figure 1 shows the type of illnesses reported by the study participants that prompted them for self-medication. The major illnesses reported were headache or fever (20.7%), GI disease (17.3%), RTI (15.9%), eye disease (14.0%), skin disease/injury (13.1) and dysmenorrhea (11.3%).

Type of illnesses reported by the study participants that prompted them for self-medication is shown in Figure 1. The major illnesses reported were headache or fever (20.7%), gastrointestinal (GI) disease (17.3%), respiratory tract infection (RTI) (15.9%), eye disease (14.0%), skin disease/injury (13.1) and dysmenorrhea (11.3%).

The major reasons listed by the self-medicated study participants are shown in Table 2. Self-medicated respondents provided reasons that 21.7% of them believed the disease was not serious; 20.7% of them have had prior experience to the illness and/or the drug; 20.2% of them were of the opinion that it was less expensive in terms of time and money; 17.0% of them believed that it was an emergency care; and 16.9% of them requested medications for prevention of known or unknown illness or symptoms of illnesses.

**Table 2:** Reasons for self-medication (n = 270).

Reason for self-medication	Frequency	Percentage
Emergency use	123	17.0
Disease not serious	157	21.7
For prevention of known or unknown disease	122	16.9
Prior experience of the drug	150	20.7
Less expensive in terms of time and money	146	20.2
Others	26	3.6

Following the reasons for self-medication, drug consumers were asked or observed on the types of drugs they requested. The majority (31.9%) of the drug consumers made their requests by telling their symptoms to a pharmacy professional. One hundred and twenty (20.1%) of the respondents requested drugs by mentioning specific names of the drugs or drug products, generic or brand while 5.0% of them by mentioning the category of the drug to which it belonged. However, 18.8% of the drug consumers were requesting drugs by showing old samples or packages of drug products, 12.6% by presenting pieces of paper and 7.7% by describing the physical characteristics such as the color and/or shape of drug products (Table 3).

**Table 3:** Type of requests for self-medication (n = 270).

Type of requests for the drug	Frequency	Percentage
Mentioning the name of the drug	120	20.1
Mentioning the category of the drug	30	5.0
Telling the symptoms of illness	190	31.9
Showing an old sample/package of the drug	112	18.8
Presenting piece of paper (not a prescription)	75	12.6
By describing physical characteristics of the drug	46	7.7
Others	23	3.9

**Figure 2:**
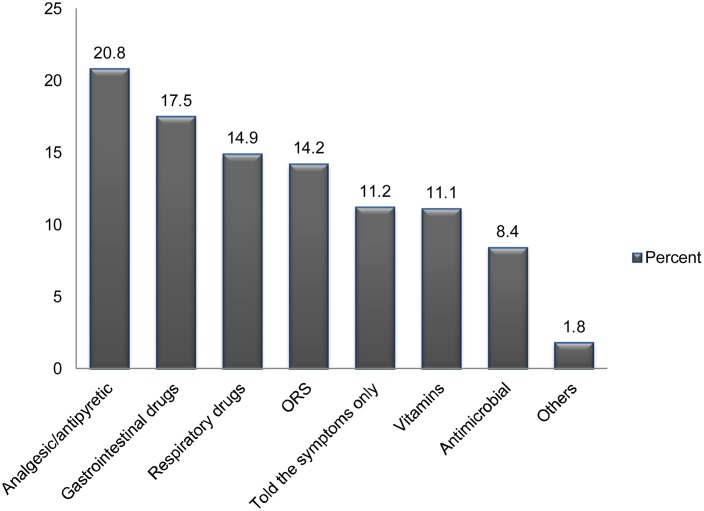
Category of drugs requested for self-medication (n = 270). Figure 2 demonstrates the most frequently requested category of drugs by self-medicated respondents. The most commonly requested category of drugs were analgesics/antipyretics (20.8%), GI drugs (17.5%), respiratory drugs (14.9%), ORS (14.2%), vitamins (11.1%) and antimicrobials (8.4%).

The most often requested category of drugs by self-medicated respondents were analgesics/antipyretics (20.8%), GI drugs (17.5%), respiratory drugs (14.9%), oral rehydration salt (ORS) (14.2%), vitamins (11.1%) and antimicrobials (8.4%) (Figure 2).

The three most usual sources of advice/information for self-medication were pharmacists (22.9%), healthcare providers such as doctors, nurses and health assistants, but without formal prescriptions (20.6%), and friends, neighbors or relatives (18.5%). Nevertheless, 12.8% of the respondents obtained information by reading drug related materials such as labels, leaflets or promotional materials while 12.5% of them were suggested by traditional healers (Table 4).

**Table 4:** Sources of advice for self-medication (n = 270).

Sources of advice for self-medication	Frequency	Percentage
Received no information	69	9.9
Read information material of the drug	89	12.8
Advised by neighbors, friends or relatives	129	18.5
Suggested by traditional healers	88	12.6
Advised by physicians, nurses and health assistants but without prescription	144	20.6
Recommended by pharmacists or those working in the pharmacy	160	22.9
Others	19	2.7

## Discussion

Self-medication gives more freedom to patients in taking care of minor ailments. It makes consumer more health conscious, reduces treatment burden on healthcare facilities and curtails the cost and time of gaining access to treatment. On the other hand, self-medication increases risks such as excessive use of medication, extended duration of consumption, incorrect diagnosis, drug interactions and polypharmacy [10].

In this study, analgesics/antipyretics were the most commonly used class of drugs as reported in other studies [11]–[15]. This is because such drugs are applied to treat uncomplicated common illness such as headache, fever and pain. This confirms that analgesics/antipyretics are the commonly used over-the-counter medications for self-medication [16]. Over-the-counter drugs, selected and utilized properly, can be highly effective in ameliorating symptoms while avoiding trivial or unnecessary physician office visits and more expensive, but not always more effective, prescription drug use [7]. Similarly GI and respiratory drugs were usually utilized for the treatment of GI diseases and respiratory infections in the present work as indicated in other studies [12], [13]. Requests for ORS, a lifesaving OTC drug, were eminent for all reported illnesses. This contradicts with the results of other studies where requests for ORS were very low for ORS [12], [13]. Th may have resulted from high public awareness on the usefulness of ORS in the management of diarrheal diseases.

On the other hand, antibiotic self-medication needs a special attention. Self-medication with other drugs can cause harm only to the person who consumes it; however, antibiotic self-medication have a global risk of spread of antibiotic resistance [5]. High prevalence of self-medication with antibiotics was widely reported in many studies [12], [13], [17]–[19]. Nevertheless, requests for antimicrobial drugs were very low for all reported illnesses in this study. The possible explanations for the lower in our work might be that there is a public awareness of the problems associated with antibiotic self-medication.

Non-seriousness of the illness, prior experience and less expensive in terms of time and money were the major reasons for self-medication in this survey. These reasons for self-medication were frequently reported in other studies [9], [11], [16], [18]. Most of the drug consumers made their requests by telling their symptoms, by mentioning specific names of the drugs and by showing old samples or packages of drug products as reported in other studies [12], [13]. Major information sources for self-medication were pharmacists followed by other healthcare providers such as physicians, nurses and health assistants in this study. Pharmacists can play a crititcal role in assisting people to make informed self-care choices. According to the charter of collaboration between the Pharmaceutical Group of the European Community and the European Proprietary Medicines Manufacturers’ Association: “The pharmacist is an adviser to the public on everyday healthcare and is a key figure in the supply and delivery of medicines to the consumer. He is a partner of the manufacturer of non-prescription medicines. Both share the common goals of service of high quality for the patient and encouragement of the rational use of medicines. The pharmacist in his professional capacity and in direct contact with patients is competent to provide sound advice on the medicines he supplies” [2]. Hence the role of community pharmacists in self–medication needs to be encouraged to promote responsible self-medication.

## Conclusions

People of all socio-demographic categories practice self-medication. The most frequent illnesses or symptoms of illnesses were headache/fever, gastrointestinal diseases and respiratory tract infections. Non-seriousness of the illness, prior experience and less expensive were cited to be the major reasons for self-medication. The majority of drug consumers made their requests by telling their symptoms of illness, by mentioning specific names of the drugs and showing old samples or packages of drug products. Analgesics/antipyretics, GI drugs, respiratory drugs and ORS were the most frequently requested category of drugs by self-medicated respondents while requests for antibiotics were very low. These findings demonstrated that self-medication practices were common in a wide range of illnesses. Health professionals, especially community pharmacists need to educate people on the benefits and risks of self-medication to encourage responsible self-medication.
